# Multicolor Mechanochromic Polymer Blends That Can Distinguish between Tensile–Stress States

**DOI:** 10.1002/marc.202400812

**Published:** 2024-12-04

**Authors:** Kuniaki Ishizuki, Akira Takahashi, Hideyuki Otsuka

**Affiliations:** ^1^ Department of Chemical Science and Engineering Institute of Science Tokyo 2‐12‐1 Ookayama, Meguro‐ku Tokyo 152‐8550 Japan; ^2^ Research Center for Autonomous Systems Materialogy (AsMAT) Institute of Integrated Research Institute of Science Tokyo 4259 Nagatsuta‐cho, Midori‐ku, Yokohama, Kanagawa Tokyo 226‐8501 Japan

**Keywords:** mechanochemistry, mechanochromism, polymer blends, polymer reactions, radicals

## Abstract

Mechanochromic polymers can be used to visualize mechanical stimuli applied to materials. However, many of these polymers exhibit single‐color mechanochromism, i.e., the polymer changes from its original color to another, thus potentially limiting the range of applications. Here, a versatile and readily accessible strategy for creating multicolor mechanochromic polymer blends that can detect whether a material is currently under stress or has already experienced stress is presented. The polymer blends are prepared by blending a segmented polyurethane and a polycaprolactone, each embedded with a radical‐type mechanochromophore. These polymers appear either blue, pink, or green by stretching depending on the mechanochromophore employed. The introduction of different mechanochromophores into each of the segmented polyurethane and polycaprolactone polymers with different chain mobilities and the subsequent blending of these polymers affords a mechanochromic polymer blend that can be used to visually distinguish via a color change whether it is experiencing stress or has recently experienced stress. The colors observed under stress and after stress can be tuned as easily as mixing paint based on the combination of the mechanochromophores (“rainbow mechanochromism”). The strategy developed in this study can be expected to significantly advance the research of mechanochromic polymer materials.

## Graphical abstract

Mechanochromic polymers that can change color in response to mechanical stimuli have attracted much attention. Here, multicolor mechanochromic polymer blends are reported that can distinguish stretching states by different colors. Moreover, the polymer blends also demonstrated applicability based on rainbow mechanochromism which allows color tuning freely and easily based on three mechanochromophores that show blue, pink, and green color changes.

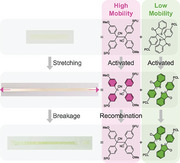

## 1 Introduction

Mechanochromic polymers, which can change color in response to mechanical stimuli, have been extensively investigated in recent years.^[^ ^1^, ^2^, ^3^, ^4^, ^5^, ^6^, ^7^, ^8^, ^9^, ^10^, ^11^, ^12^, ^13^, ^14^ ^]^ Mechanochromic polymers can be used to visualize molecular‐scale deformation and/or breakage and are expected to find applications in various smart materials, such as those with damage‐visualization capabilities. One strategy to design mechanochromic polymers is to introduce mechanochromophores, i.e., mechanically sensitive molecules that change color in response to mechanical stimuli, into polymer structures. Ever since the publication of a pioneering study on mechanochromic polymers containing spiropyran in the center of the polymer chain,^[^ ^3^ ^]^ there have been various reports on mechanochromic polymers based on different elaborately designed mechanochromophores.^[^ ^4^, ^5^, ^6^, ^7^, ^8^, ^9^, ^10^, ^11^, ^12^, ^13^, ^14^ ^]^

Importantly, most mechanochromic polymers exhibit only a simple color change from one color to another. This is known as single mechanochromism and can be used to detect whether a material has experienced stress, but it is not suitable to visualize a potentially more complex stress history. On the other hand, mechanochromic polymers that show multiple color changes, i.e., multicolor mechanochromic polymers, have attracted much attention as they can realize more complex color changes depending on the history of the applied external stimuli.^[^ ^15^, ^16^, ^17^, ^18^, ^19^, ^20^, ^21^, ^22^, ^23^, ^24^, ^25^, ^26^ ^]^ One strategy to design multicolor mechanochromic polymers is based on using a mechanochromophore that can express multiple mechanochromic responses.^[^ ^15^, ^16^, ^25^ ^]^ Such mechanochromic polymers have been accomplished with polymers that contain, e.g., spiropyran^[^ ^15^ ^]^ or rhodamine.^[^ ^16^ ^]^ However, it is difficult to design mechanochromophores that undergo multiple color changes within a single molecule. In addition, it is even more challenging to design molecules that flexibly express the desired shades of color.

To this end, using multiple mechanochromophores is a more feasible and versatile strategy.^[^ ^17^, ^18^, ^19^, ^20^, ^21^, ^22^, ^23^, ^24^, ^26^ ^]^ While one can use mechanochromophores with different mechanochromic properties,^[^ ^18^, ^19^, ^22^, ^23^, ^24^, ^26^ ^]^ a more versatile protocol would introduce different mechanochromophores into environments within the material that exhibit varying stress behavior.^[^ ^17^, ^18^, ^20^, ^21^ ^]^ By introducing different mechanochromophores into different polymer networks, Zhang et al. have demonstrated a multicolor mechanochromic multi‐network elastomer that can detect the intensity of mechanical stimuli.^[^ ^21^ ^]^ Our group has also reported a multicolor mechanochromic polymer/silica composite with two different mechanochromophores in the polymer domain and at the interface of the polymer and silica domains, respectively.^[^ ^18^ ^]^ However, these examples require a specific polymer design and therefore synthetic method, such as the double‐network formation or the sol‐gel method, which severely limits the scope of these materials.

**Figure 1:**
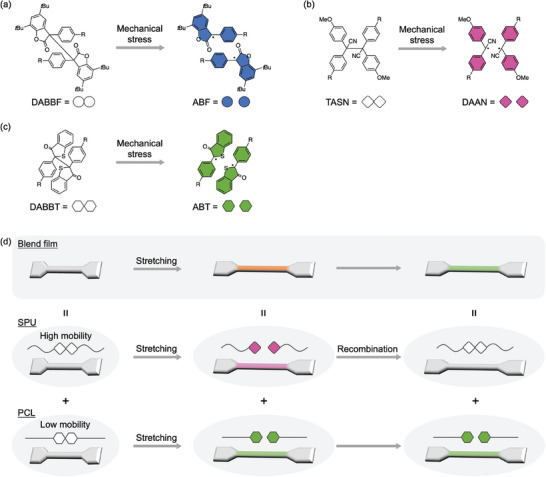
Chemical structures of a) DABBF, b) TASN, and c) DABBT before and after applying mechanical stress. DABBF‐diol, TASN‐diol, and DABBT‐diol are the diol derivatives of the mechanochromophores, where ─R = ─OCH_2_CH_2_CH_2_OH. d) Schematic illustration of the mechanochromic and discoloration behavior of SPU, PCL, and their blend films governed by the chain mobility.

On the other hand, multicolor mechanochromic polymers made from polymer blends do not require such a specific polymer design, making them more versatile and easier to synthesize. Our group has extensively developed radical‐type mechanochromophores such as diarylbibenzofuranone (DABBF),^[^ ^7^, ^18^, ^28^, ^29^, ^30^, ^31^, ^32^ ^]^ tetraarylsuccinonitrile (TASN),^[^ ^33^, ^34^, ^35^ ^]^ and diarylbibenzothiophenonyl (DABBT),^[^ ^27^ ^]^ which undergo homolytic cleavage of the central carbon─carbon bond in response to mechanical stress resulting in colored radicals, i.e., arylbenzofuranonyl (ABF, blue), diarylacetonitrile (DAAN, pink), and arylbenzothiophenonyl (ABT, green) (**Figure** **1**a–c). As these mechanochromophores exhibit a similar mechanoresponsiveness, we were able to demonstrate that the mechanically induced color can be easily tuned by mixing these mechanochromophores like paint, a process which has been coined “rainbow mechanochromism.”^[^ ^27^ ^]^ Using this strategy, we have achieved multicolor mechanochromic polymer blends that can distinguish different types of mechanical stimuli (grinding or stretching)^[^ ^17^ ^]^ and the duration of the applied stress^[^ ^20^ ^]^ by introducing two different mechanochromophores into different environments in the polymer blends. While these studies are mainly based on grinding systems, the demonstration of the effectiveness of multicolor mechanochromism under other types of mechanical stresses and the establishment of general synthetic guidelines is strongly required for the further development of mechanochromic polymer materials.

We herein report multicolor mechanochromic polymer blends based on a bulk system with multiple mechanochromophores introduced into polymers with different mobilities. We designed polymer blends consisting of segmented polyurethanes (SPUs) and polycaprolactones (PCL) that each contain different mechanochromophores. When the radical‐type mechanochromophores are cleaved, the resulting color derived from the stable radicals afforded by the cleavage will fade due to radical recombination on a timescale that depends on the polymer chain mobility around the embedded mechanochromophores. Mechanochromic SPU can show a time‐limited coloration. On the other hand, the chain mobility of PCL is restricted by the crystalline region, i.e., the inner mechanochromophore can maintain the coloration much longer than SPU. When these two polymers are blended and subjected to stress, it was expected that the blends will initially show the combined color of the mechanically formed radicals from both the SPU and PCL (Figure 1d). However, after the stress is removed, the color from the SPU will soon fade due to radical recombination caused by the high chain mobility of SPU. Conversely, the PCL‐derived color will be retained for longer due to the restricted chain mobility of PCL, which inhibits recombination of the colored radicals. Thus, the rainbow mechanochromism created by stretching is applied to multicolor mechanochromic polymer blends that can, via the introduction of two mechanochromophores into polymer chains with different mobilities, distinguish different stretching states. This difference in the degree of recombination is expected to afford a different color during and after the application of stress.

## 2 Results and Discussion

**Figure 2:**
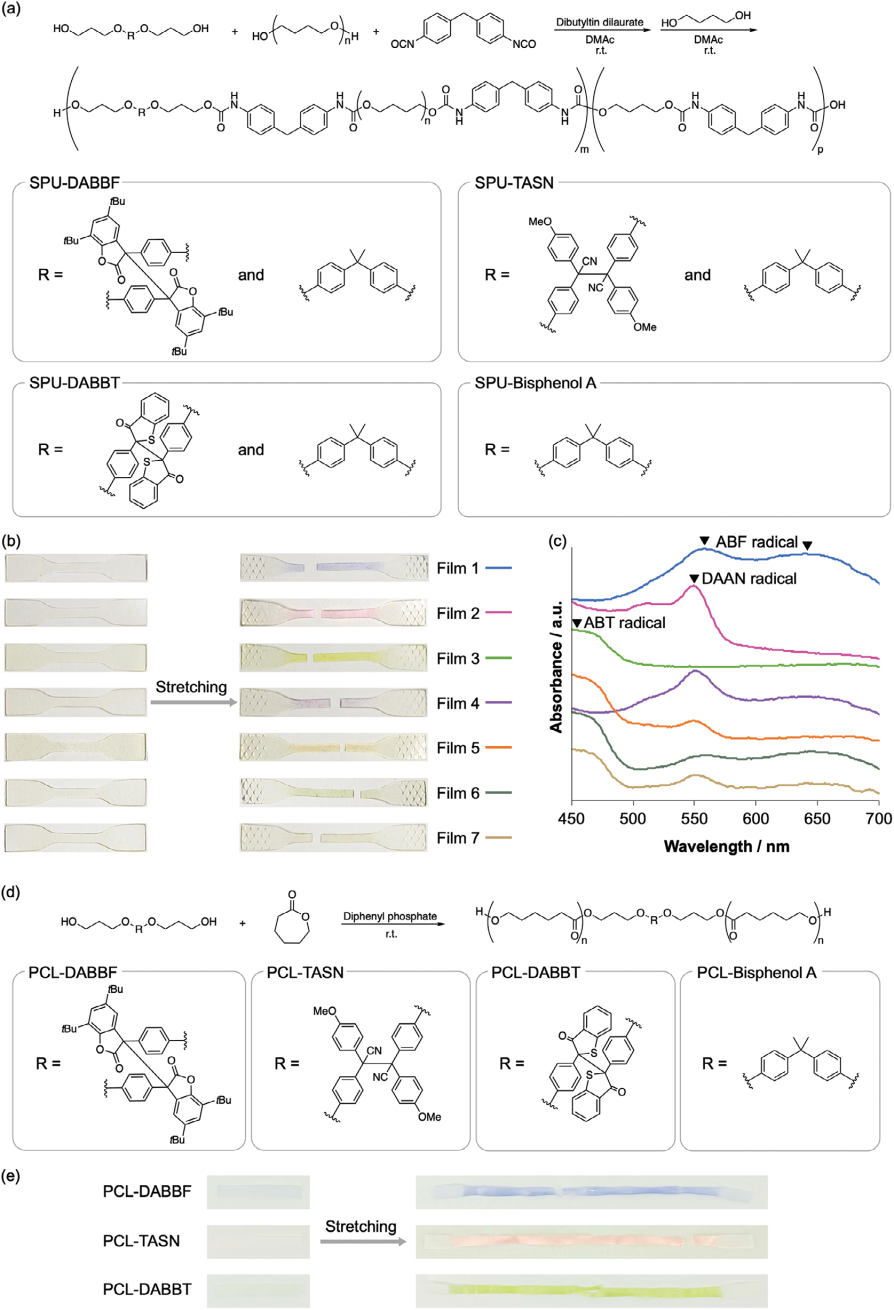
a) Synthesis of the SPU polymers containing DABBF, TASN, DABBT, and bisphenol A. b) Photographs of Films 1–7 before and after stretching. c) Solid‐state UV/vis spectra of Films 1–7 after stretching. d) Synthesis of PCL polymers that contain DABBF, TASN, DABBT, and bisphenol A. e) Photographs of PCL‐DABBF, PCL‐TASN, and PCL‐DABBT films before and after stretching.

Mechanochromic SPU polymers that incorporate either DABBF (SPU‐DABBF), TASN (SPU‐TASN), or DABBT (SPU‐DABBT) were synthesized via a prepolymer method. The mechanochromophores were introduced into the soft segments of the SPU to gain molecular mobility. The molecular mobility can be adjusted by the hard segments, i.e., by forming hydrogen bonds via the urethane linkages and functioning as physical crosslinking points, thus helping the efficient transmission of the external force to the molecular chain. We initially synthesized the SPU polymers with hard‐segment‐rich compositions (SPU‐H). We chose to use poly(tetramethylene ether glycol) (PTMG) as the polymeric diol for the SPU synthesis because when PTMG is mixed with PCL, the resulting mixture is known to form a dispersed phase‐matrix structure with a phase domain.^[^ ^36^ ^]^ The SPU polymers were synthesized first by forming the soft segments from the diol derivatives of each mechanochromophore (DABBF‐diol, TASN‐diol, or DABBT‐diol), PTMG (*M*_n_ = 1000), and 4,4’‐methylenebis(phenyl isocyanate) (MDI), followed by the addition of 1,4‐butanediol (BDO) as the chain extender (**Figure** **2**a). The structures of the synthesized SPU‐Hs were characterized using ^1^H NMR spectroscopy, gel permeation chromatography (GPC), and differential scanning calorimetry (DSC) (for details, see the Supporting Information), and the results are summarized in **Table** **1**. The relatively small *M*_n_ of SPU‐H is presumably due to the presence of many urethane bonds resulted from its hard‐segment‐rich composition, making the hydrodynamic radius small. DSC measurements showed that the *T*_g_ of the soft segment (*T*_g1_) is well below room temperature for all the SPU polymers, indicating that the mechanochromophores embedded in the soft segments can be expected to have good mobility as designed. The *T*_g_ of the hard segments (*T*_g2_) was observed above room temperature, indicating that the chain mobility is, at least to an extent, restricted by the hard segment.

**Table 1:** The synthesis and resulting properties of the obtained SPU polymers.

	PTMG / BDO [mol]	Yield [%]	*M*_n_ [g mol^−1^]	*M*_w_ / *M*_n_	*T*_g1_ [°C]	*T*_g2_ [°C]
SPU‐DABBF‐H	1.0/2.0	94	4,880	4.40	−19.6	29.2
SPU‐TASN‐H	1.0/2.1	96	5,120	3.26	−25.6	44.0
SPU‐DABBT‐H	1.0/2.1	92	6,140	3.45	−17.6	45.3
SPU‐DABBF‐S	1.0/0.60	85	15,220	3.21	−12.7	–
SPU‐TASN‐S	1.0/0.70	80	14,220	4.92	−21.5	–
SPU‐Bisphenol A‐S	1.0/0.61	97	12,220	3.99	−19.9	–

To confirm the intrinsic mechanochromic properties of the obtained SPU‐DABBF‐H, SPU‐TASN‐H, and SPU‐DABBT‐H polymers, a film sample of each was prepared via a solvent‐casting method (Films 1–3). The color tunability of the SPU polymers was also investigated by simply mixing each SPU with another SPU with a different mechanochromophore (Films 4–7, **Table** **2**). When Films 1–3, which were composed each of a single SPU, were stretched, they all showed the intrinsic color of the embedded mechanochromophore: blue for DABBF, pink for TASN, and green for DABBT (Figure 2b). On the other hand, the blend films showed a color change different from any of the individual polymers, indicative of the appearance of a mixed color depending on the combination of different mechanochromophores. For example, Film 4, which contained SPU‐DABBF‐H and SPU‐TASN‐H, exhibited a purple color change upon stretching, which is believed to arise from the mixed color derived from the ABF and DAAN radicals. Films 1–7 exhibited similar stress–strain curves (Figure S17, Supporting Information), showing their comparable mechanical properties.

**Table 2:** Compositions of the SPU films.

Film	Polymer blend^a)^
1	SPU‐DABBF‐H
2	SPU‐TASN‐H
3	SPU‐DABBT‐H
4	SPU‐DABBF‐H / SPU‐TASN‐H
5	SPU‐TASN‐H / SPU‐DABBT‐H
6	SPU‐DABBT‐H / SPU‐DABBF‐H
7	SPU‐DABBF‐H / SPU‐TASN‐H / SPU‐DABBT‐H

^a)^ Weight ratios of the SPU polymers are identical.

Solid‐state UV/vis measurements of the stretched Films 1–3 showed the maximum absorption corresponding to each radical species derived from the corresponding mechanochromophore, i.e., 550 nm and 640 nm for the ABF radical, 540 nm for the DAAN radical, and 450 nm for the ABT radical (Figure 2c). The spectrum of Film 4 was attributed to the sum of the absorption bands of the ABF and DAAN radicals. Similarly, Film 5, which is composed of SPU‐TASN‐H and SPU‐DABBT‐H, exhibited an orange color change and solid‐state UV/vis spectrum that corresponds to the sum of the absorption spectra of the DAAN and ABT radicals. The blend of SPU‐DABBT‐H and SPU‐DABBF‐H (Film 6) and that of all three polymers (Film 7) also afforded similar results. These results show that each mechanochromophore in the blend films undergoes sufficient dissociation under stretching to make its color visible, thus supporting the feasibility of creating targeted multicolor mechanochromism based on the polymer blend.

Based on the aforementioned results, we next synthesized SPU polymers with a lower percentage of the hard segment (SPU‐S) as the primary samples to be used to achieve the targeted multicolor mechanochromism. The SPU‐S samples were synthesized in a manner similar to that of their SPU‐H counterparts, except that the feed ratio of BDO was reduced to about one‐third compared to the SPU‐H polymers. A SPU‐S polymer containing bisphenol A instead of the mechanochromophores (SPU‐Bisphenol A‐S) was also synthesized for reference purposes. DSC measurements showed that the *T*_g1_ for the soft segments is well below room temperature for all SPU‐S polymers examined; however, the measurements did not show a clear *T*_g2_ for the hard segments on account of the decrease in the hard‐segment ratio. As expected, the obtained SPU‐S polymers exhibited a short‐lived mechanochromic response upon stretching (it quickly faded within 2 min; Figure S28, Supporting Information), indicative of the high chain mobility that facilitates recombination of the formed colored radicals.

Next, mechanochromic PCL polymers were synthesized for use as low‐mobility polymers to be used in the multicolor mechanochromic polymer blends that can distinguish stretching states. The PCL polymer was designed to incorporate the mechanochromophores at the center of the polymer chain to most efficiently utilize the mechanochromic properties. The targeted PCL polymers were synthesized via the ring‐opening polymerization of ε‐caprolactone using the diol derivatives of each mechanochromophore (DABBF‐diol, TASN‐diol, or DABBT‐diol) or bisphenol A‐diol as the initiators in the presence of a diphenyl phosphate catalyst (Figure 2d). The synthesized PCL polymers were structurally characterized using ^1^H NMR spectroscopy, GPC, and DSC (for details, see the Supporting Information), and the results are summarized in **Table** **3**. All DSC measurements showed the *T*_m_ at 53.8 °C and the *T*_g_ at around –61 °C for all the PCL samples. The PCL polymers with the embedded mechanochromophores showed mechanochromism upon stretching, whereby the color change depends on the introduced mechanochromophore (Figure 2e). Importantly, the color did not fade, not even after 24 h (Figure S29, Supporting Information). These results clearly highlight that the obtained PCL polymers are responsive to tensile stress imparted on their flexible amorphous regions and that they have the ability to suppress the recombination of the generated colored radicals due to the presence of the crystalline regions.

**Table 3:** The synthesis and resulting properties of the obtained PCL polymers.

	Yield [%]	*M*_n_ [g mol^−1^]	*M*_w_/*M*_n_	*T*_g_ [°C]	*T*_m_ [°C]
PCL‐DABBF	61	81,800	1.10	−60.8	53.8
PCL‐TASN	88	86,600	1.73	−61.0	53.8
PCL‐DABBT	43	86,200	1.18	−60.5	53.8
PCL‐Bisphenol A	54	84,500	1.10	−60.7	53.8

**Figure 3:**
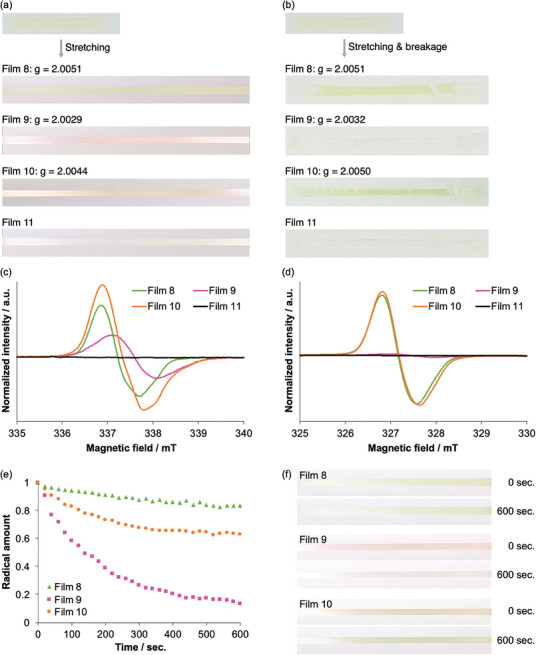
Photographs of Films 8–11 a) during tensile tests and b) after breakage. EPR spectra of Films 8–11 c) during tensile tests and d) after breakage. e) Change in the stretching‐induced amounts of radicals in Films 8–10 as a function of time. f) Photographs of Films 8–10 at 0 and 600 s after application of constant strain.

With the mechanochromic SPU and PCL polymers in hand, we then moved on to the development of the targeted multicolor mechanochromic polymer blends that can distinguish different stretching states. The blend films were prepared via a solvent‐casting method with a tetrahydrofuran solution of each SPU‐S and PCL sample (**Table** **4**). The *T*_g_ of Film 10 was −39.1 °C (Figure S30, Supporting Information), which is between those of PCL‐DABBT and SPU‐TASN‐S, suggesting that they are miscible in the amorphous region. The results are summarized in **Figure** **3**. When Film 8, which only contained the DABBT mechanochromophore in the PCL polymer, was stretched, a long‐lasting green mechanochromic response derived from the ABT radical was observed, suggesting that the recombination of the formed ABT radicals is restricted, even in the blend film. In contrast, stretching of Film 9, which only contained the TASN mechanochromophore in the SPU polymer, resulted in a temporary pink mechanochromic response derived from the DAAN radicals which soon faded after breakage. This behavior is indicative of the quick recombination of the DAAN radicals due to the high chain mobility around the TASN group in the blend film. These results are consistent with those of the stretching tests conducted on either PCL or SPU films alone, showing that the mechanochromic properties of the PCL and SPU polymers are retained, even in the blend films. Based on these results, we then blended PCL and SPU polymers that contained DABBT and TASN mechanochromophores, respectively (Film 10). Film 10 exhibited a stress–strain curve intermediate between SPU‐TASN‐S and PCL‐DABBT (Figure S31, Supporting Information). During stretching, the films exhibited an orange coloration similar to Film 5 (Figure 2b), indicative of the formation of both ABT and DAAN radicals. However, only the green color remained following breakage of the film, suggesting that the DAAN radicals preferentially recombined due to their higher mobility in the SPU polymer. A control experiment was carried out using a blend film without any mechanochromophores (Film 11), and no color change was observed either during or after stretching.

**Table 4:** Compositions of the SPU and PCL blended films.

Film	Polymer blend^a)^
8	SPU‐Bisphenol A‐S / PCL‐DABBT
9	SPU‐TASN‐S / PCL‐Bisphenol A
10	SPU‐TASN‐S / PCL‐DABBT
11	SPU‐Bisphenol A‐S / PCL‐Bisphenol A
12	SPU‐TASN‐S / PCL‐DABBF
13	SPU‐DABBF‐S / PCL‐DABBT
14	SPU‐DABBF‐S / PCL‐TASN

^a)^ Weight ratio of SPU and PCL is 3/5 in each case.

To investigate the mechanochromism in more detail, electron paramagnetic resonance (EPR) measurements were conducted. Simultaneous EPR measurements during the ‐stretching of Films 8–10 showed the generation of radicals (Figure 3c). The *g* values of the radicals from Film 8 (2.005) and Film 9 (2.003) were attributed to the ABT and DAAN radicals, respectively.^[^ ^27^, ^33^ ^]^ On the other hand, the *g* value of the radicals from Film 10 (2.004) is an intermediate value between those of the ABT and DAAN radicals, and thus suggests that both radicals were formed. Next, we conducted the EPR measurements 10 min after breakage of the films. Film 8 and Film 10 showed radicals with a *g* value of 2.005, attributed to the ABT radicals. Meanwhile, Film 9 showed a very small amount of radicals with a *g* value of 2.003, attributed to the DAAN radicals (Figure 3d), showing that the DAAN radicals in the SPU polymer were rapidly recombined after breakage of the film.

To quantitatively evaluate the radical recombination behavior after stretching, EPR measurements were carried out over a set time period to monitor the quantity of radicals generated over time after the stretching of Films 8–10 at 250% elongation. We found that the decay rate followed the order, from fastest to slowest, Film 9 > Film 10 > Film 8 (Figure 3e). Film 8 with the DABBT mechanochromophore in the PCL polymer retained 83% of the initially formed radicals and remained green, even after stretching for 600 seconds (Figure 3f). In contrast, the amount of radicals in Film 9 with the TASN mechanochromophore in the SPU polymer, when stretched for 600 seconds dropped to 13%, which is about 6 times lower than the amount of radicals in Film 8. Consequently, the pink color of Film 9 had almost faded after 600 seconds (Figure 3f). Film 10, with the DABBT mechanochromophore in the PCL polymer and the TASN mechanochromophore in the SPU polymer, showed an intermediate decay rate, where 63% of the initially formed radicals remained after stretching. Considering the nature of the color change from orange at 0 seconds to green at 600 seconds, the remaining radicals at 600 seconds were attributed to the ABT radicals (Figure 3f). This is further supported by the similarity of the EPR spectra of Film 10 and Film 8 taken 10 min after stretching and breakage (Figure 3d). These results quantitatively demonstrate that the DAAN radicals in the SPU polymer recombine faster than the ABT radicals in the PCL polymer due to the differences in chain mobility.

**Figure 4:**
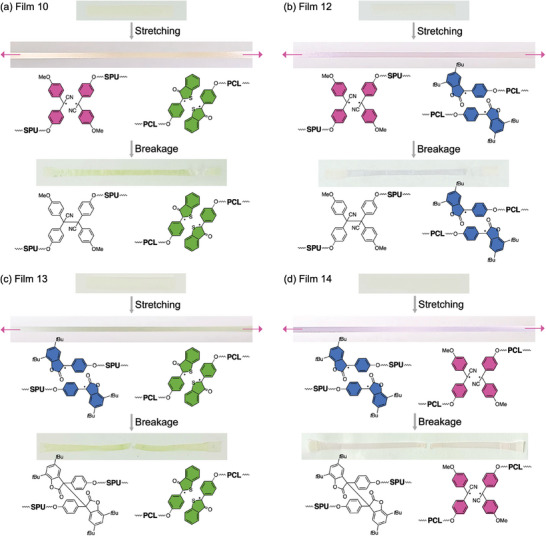
Photographs of a) Film 10, b) Film 12, c) Film 13, and d) Film 14 during and after tensile tests.

The color of the film during and after stretching can be freely adjusted depending on which mechanochromophore is introduced. For example, Film 12, obtained by blending SPU‐TASN‐S and PCL‐DABBF, exhibited a purple color during stretching and a blue color after breakage (**Figure** **4**b). During the stretching, both the blue ABF and pink DAAN radicals were present, but only the blue ABF radicals remained after the breakage due to the rapid recombination of the DAAN radicals. Similarly, Film 13, obtained by blending SPU‐DABBF‐S and PCL‐DABBT, showed a dark green color during stretching derived from both the blue ABF and green ABT radicals, but only the green ABT radicals remained after breakage (Figure 4c). Film 14 composed of SPU‐DABBF‐S and PCL‐TASN, which are the opposite combination of mechanochromophore and polymer to Film 12, showed a purple color derived from both the blue ABF and pink DAAN radicals, as observed in Film 12 during stretching, but a pink color was seen after breakage (Figure 4d) due to the rapid recombination of the ABF radicals. The above results show that our rainbow mechanochromism approach based on a library of radical‐type mechanochromophores enables the design of diverse color expressions in response to different mechanical states of multicolor mechanochromic polymer blends.

## 3 Conclusion

In this paper, we have demonstrated multicolor mechanochromic polymer blends that can be used to visualize the stress application state of the blend through coloring of a desired color. A library of our radical‐type mechanochromophores, that can exhibit either green, pink, or blue colors depending on the molecule used, enables color‐tunable mechanochromism (“rainbow mechanochromism”), as confirmed when using films of segmented polyurethane (SPU) polymers with hard‐segment‐rich compositions (SPU‐H). The introduction of different mechanochromophores into SPU and polycaprolactones (PCL) polymers followed by mixing of these polymers led to polymer blends that show different colors during and after stretching. Furthermore, the color of each of these states can be tuned on demand based on various combinations of the mechanochromophores. The strategy of this study is not limited to the combination of SPU and PCL polymers but can be applied to a wide variety of polymer combinations with different chain mobilities. The results obtained here represent a big step toward the practical application of mechanochromic polymers.

## Conflict of Interest

The authors declare no conflict of interest.

## Supporting information



Supporting Information

## Data Availability

The data that support the findings of this study are available in the supplementary material of this article.
