# Supplementary material for: Multicolor Mechanochromic Polymer Blends That Can Distinguish between Tensile–Stress States
Source: Macromol Rapid Commun. 2024 Dec 4;46(6):2400812. doi: 10.1002/marc.202400812 (PMC11925322; doi:10.1002/marc.202400812)
Supplement: Supplementary file 1 — Supporting Information [file MARC-46-2400812-s001.docx]

Supporting Information

Multicolor Mechanochromic Polymer Blends That Can Distinguish between Tensile-Stress States

Kuniaki Ishizuki,^[a]^ Akira Takahashi,^[a]^ and Hideyuki Otsuka*^[a,b]^

[a] K. Ishizuki, Dr. A. Takahashi, and Prof. H. Otsuka

Department of Chemical Science and Engineering, Institute of Science Tokyo

2-12-1 Ookayama, Meguro-ku, Tokyo 152-8550, Japan

E-mail: otsuka@mac.titech.ac.jp

[b] Prof. H. Otsuka

Research Center for Autonomous Systems Materialogy (ASMat),
Institute of Integrated Research, Institute of Science Tokyo

**Materials**

All solvents and reagents were purchased from Sigma-Aldrich, FUJIFILM Wako Pure Chemical Corporation, Tokyo Chemical Industry, and Kanto Chemical and used as received, unless otherwise noted. 1,4-Butanediol and ε-caprolactone were distilled under reduced pressure. DABBF-diol^1)^, TASN-diol^2)^, DABBT-diol^3)^, PCL-TASN^4)^, and PCL-Bisphenol A^4)^ were synthesized according to previously published methods.

**Instruments**

Gel permeation chromatography (GPC) was carried out at 40 °C on Tosoh HLC-8320 GPC system equipped with a guard column (Tosoh TSK guard column Super H-L), three columns (Tosoh TSK gel SuperH 6000, 4000, and 2500), a differential refractive index detector, and a UV/vis detector. Tetrahydrofuran (THF) was used as the eluent at a flow rate of 0.6 mL/min. Polystyrene (PS) standards (*M*_n_ = 4430–3242000; *M*_w_/*M*_n_ = 1.03–1.08) were used to calibrate the GPC system. Differential scanning calorimetry (DSC) measurements were carried out using a SHIMADZU DSC-60A Plus with a heating rate of 10 °C/min. ^1^H NMR spectra were recorded on a Bruker AVANCE III HD500 spectrometer. Solid-state UV/vis measurements were performed on a JASCO V-650 spectrophotometer at r.t.

**Film Preparation**

1. SPU-H films (Films 1–7): Each of SPU-DABBF-H, SPU-TASN-H, SPU-DABBT-H, and the mixture was dissolved in tetrahydrofuran, respectively. The solutions were dried slowly on a Teflon plate for 1 day under a nitrogen atmosphere. The film was fabricated into ISO 37-4 specimens (dumbbell shape, 12 mm, 2 mm, 0.2–0.5 mm). The specimens were dried under vacuum at r.t. for 3 days.

2. SPU-S film: SPU-TASN-S was dissolved in tetrahydrofuran. The solutions were dried slowly on a Teflon plate for 12 h under a nitrogen atmosphere and dried under vacuum at r.t. for 12 h. The films were cut into 5.0 mm wide strips.

3. PCL films: Each of PCL-DABBF, PCL-TASN, and PCL-DABBT was dissolved in tetrahydrofuran, respectively. The solutions were dried slowly on a Teflon plate for 12 h under a nitrogen atmosphere and dried under vacuum at r.t. for 12 h. The films were cut into 5.0 mm wide strips.

4. Blend films (Films 8–14): A mixture of SPU and PCL was dissolved in tetrahydrofuran. These solutions were dried slowly on a Teflon plate for 12 h under a nitrogen atmosphere and dried under vacuum at r.t. for 12 h. The films were cut into 5.0 mm wide strips.

**Measurements**

**Tensile Tests**

Tensile tests were performed with a 100 mm/min elongation rate at r.t. on a SHIMADZU EZ-L instrument equipped with a 50 N load cell. In order to more clearly show the color during stretching and after breakage, tensile tests were performed with a 200 mm/min elongation rate at r.t. for Figure 3 and Figure 4.

**EPR Spectroscopy for Films 8–11**

The stretched samples were transferred into an EPR 5 mm glass capillary, and the capillary was sealed after being degassed. EPR measurements were carried out on a JEOL JES-X320 X-band EPR spectrometer equipped with a JEOL DVT temperature controller. The spectra of stretched samples were measured using a microwave power of 0.998 mW and field modulation of 0.4 mT with a time constant of 0.03 s and a sweep rate of 1.5 mT/s at r.t. The Mn^2+^ signal was used as an auxiliary standard. The *g* value was calculated according to the following equation: *g = hν/βH* where *h* is the Planck constant, *ν* is the microwave frequency, *β* is the Bohr magneton, and *H* is the magnetic field. EPR spectra were normalized by weight of each sample.

**EPR Spectroscopy during Tensile Deformation for Films 8–11**

Electron paramagnetic resonance (EPR) measurements during tensile deformation were carried out on a JEOL JES-F A200 EPR X-band spectrometer equipped with a Baldwin tensile using strip specimens (120 mm × 3–4 mm × 0.1–0.3 mm). The effective measuring range is 43.5 mm in height. The specimens were stretched to 250 (Figure 6) and 300% (Figure 5c) strains in sequence under a strain rate of 200 mm/min. The spectra were measured at each strain at r.t. using a microwave power of 0.998 mW and field modulation of 0.4 mT with a time constant of 0.03 s and a sweep rate of 1.5 mT/s. The Mn^2+^ signal was used as an auxiliary standard. The *g* value was calculated according to the following equation: *g = hν/βH* where *h* is the Planck constant, *ν* is the microwave frequency, *β* is the Bohr magneton, and *H* is the magnetic field. EPR spectra were normalized by the initial volume of each sample.

**Synthesis**

**Synthesis of SPU-DABBF**

SPU-DABBF-H was synthesized by the following method. In a two-neck round-bottomed flask, poly(tetramethylene ether glycol) (PTMG) (*M*_n_ = 1000) (1.24 g, 1.24 mmol) was dried in vacuo for 1.5 h at 80 °C. DABBF-diol (98.0 mg, 0.124 mmol), Bisphenol A-diol (384 mg, 1.12 mmol), *N*,*N*-dimethylacetamide (29.0 mL), and 4,4’-methylenebis(phenyl isocyanate) (MDI) (1.24 g, 4.96 mmol) were added. After the mixture was bubbled with nitrogen for 20 min, di-*n*-butyltin dilaurate diluted with tetrahydrofuran (2 drops) was added to the mixture under a nitrogen atmosphere. After stirring for 9 h at room temperature, 1,4-butanediol (BDO) (0.227 g, 2.52 mmol) was added to the mixture under a nitrogen atmosphere. After stirring for 12 h at room temperature, the reaction was stopped by adding methanol (5 drops). The crude product was added to water, precipitated in THF/methanol, and dried in vacuo to give a white solid (2.99 g, 94%).

SPU-DABBF-S was synthesized by the following method. In a two-neck round-bottomed flask, PTMG (*M*_n_ = 1000) (3.32 g, 3.32 mmol) was dried in vacuo for 1.5 h at 80 °C. DABBF-diol (0.789 g, 1.00 mmol) and Bisphenol A-diol (0.811 g, 2.33 mmol) were added. After two freeze vacuum drying cycles by benzene, *N*,*N*-dimethylacetamide (150 mL), MDI (2.16 g, 8.63 mmol), and di-*n*-butyltin dilaurate diluted with tetrahydrofuran (2 drops) were added. After stirring for 27 h at room temperature, BDO (0.180 g, 2.00 mmol) was added to the mixture under a nitrogen atmosphere. After stirring for 13 h at room temperature, the reaction was stopped by adding methanol (5 drops). The crude product was added to water, precipitated in tetrahydrofuran/water, and dried in vacuo to give a white solid (6.92 g, 95%).

^1^H NMR (500 MHz, DMSO-*d*_6_): *δ* /ppm 9.34–9.71 (br, NH), 7.22–7.46 (br, aromatic), 6.88–7.22 (br, aromatic), 6.58–6.88 (br, aromatic), 4.14–4.31 (br, OC*H*_2_*C*H_2_CH_2_), 3.94–4.14 (br, OCH_2_*C*H_2_C*H*_2_, COOC*H*_2_CH_2_CH_2_CH_2_), 3.68–3.94 (br, PhC*H*_2_), 3.04–3.52 (COOCH_2_CH_2_CH_2_C*H*_2_), 1.96–2.13 (br, OCH_2_C*H*CH_2_), 1.38–1.73 (br, PhCC*H*_3_, COOCH_2_C*H*_2_CH_2_CH_2_, COOCH_2_CH_2_C*H*_2_CH_2_), 0.89–1.38 (br, tBu).

**Synthesis of SPU-TASN**

SPU-TASN-H was synthesized by the following method. In a two-neck round-bottomed flask, PTMG (*M*_n_ = 1000) (1.31 g, 1.31 mmol) was dried in vacuo for 1.5 h at 80 °C. TASN-diol (78.0 mg, 0.132 mmol), bisphenol A-diol (406 mg, 1.18 mmol), *N*,*N*-dimethylacetamide (40.0 mL), and MDI (1.32 g, 5.27 mmol) were added. After the mixture was bubbled with nitrogen for 20 min, di-*n*-butyltin dilaurate diluted with tetrahydrofuran (2 drops) was added to the mixture under a nitrogen atmosphere. After stirring for 9 h at room temperature, BDO (0.251 g, 2.79 mmol) was added to the mixture under a nitrogen atmosphere. After stirring for 12 h at room temperature, the reaction was stopped by adding methanol (5 drops). The crude product was added to water, precipitated in THF/methanol, and dried in vacuo to give a pale-yellow solid (3.19 g, 96%).

SPU-TASN-S was synthesized by the following method. In a two-neck round-bottomed flask, PTMG (*M*_n_ = 1000) (3.37 g, 3.37 mmol) was dried in vacuo for 1.5 h at 80 °C. TASN-diol (0.599 g, 1.01 mmol) and Bisphenol A-diol (0.811 g, 2.35 mmol) were added. After two freeze vacuum drying cycles by benzene, *N*,*N*-dimethylacetamide (150 mL), MDI (2.19 g, 8.75 mmol), and di-*n*-butyltin dilaurate diluted with tetrahydrofuran (2 drops) were added. After stirring for 13 h at room temperature, 1,4-butanediol (BDO) (0.182 g, 2.02 mmol) was added to the mixture under a nitrogen atmosphere. After stirring for 13 h at room temperature, the reaction was stopped by adding methanol (5 drops). The crude product was added to water, precipitated in tetrahydrofuran/water, and dried in vacuo to give a yellow solid (5.69 g, 80%).

^1^H NMR (500 MHz, DMSO-*d*_6_): *δ* /ppm 9.36–9.70 (br, N*H*), 7.23–7.48 (br, aromatic), 6.95–7.23 (br, aromatic), 6.62–6.95 (br, aromatic), 4.14–4.30 (br, OC*H*_2_*C*H_2_CH_2_), 3.93–4.14 (br, OCH_2_*C*H_2_C*H*_2_, COOC*H*_2_CH_2_CH_2_CH_2_), 3.68–3.94 (br, PhOC*H*_3_, PhC*H*_2_), 3.04–3.52 (COOCH_2_CH_2_CH_2_C*H*_2_), 1.95–2.12 (br, OCH_2_C*H*CH_2_), 1.29–1.73 (br, PhCC*H*_3_, COOCH_2_C*H*_2_CH_2_CH_2_, COOCH_2_CH_2_C*H*_2_CH_2_).

**Synthesis of SPU-DABBT**

SPU-DABBT-H was synthesized by the following method. In a two-neck round-bottomed flask, PTMG (*M*_n_ = 1000) (1.31 g, 1.31 mmol) was dried in vacuo for 1.5 h at 80 °C. DABBT-diol (74.3 mg, 0.124 mmol), Bisphenol A-diol (384 mg, 1.12 mmol), *N*,*N*-dimethylacetamide (28.5 mL), and MDI (1.25 g, 5.00 mmol) were added. After the mixture was bubbled with nitrogen for 20 min, di-*n*-butyltin dilaurate diluted with tetrahydrofuran (2 drops) was added to the mixture under a nitrogen atmosphere. After stirring for 9 h at room temperature, BDO (0.249 g, 2.76 mmol) was added to the mixture under a nitrogen atmosphere. After stirring for 12 h at room temperature, the reaction was stopped by adding methanol (5 drops). The crude product was added to water, precipitated in THF/methanol, and dried in vacuo to give a white solid (2.91 g, 92%).

^1^H NMR (500 MHz, DMSO-*d*_6_): *δ* /ppm 9.32–9.70 (br, NH), 7.47–7.78 (br, aromatic), 7.21–7.47 (br, aromatic), 6.94–7.21 (br, aromatic), 6.59–6.94 (br, aromatic), 4.14–4-31 (br, OC*H*_2_*C*H_2_CH_2_), 3.94–4.14 (br, OCH_2_*C*H_2_C*H*_2_, COOC*H*_2_CH_2_CH_2_CH_2_), 3.68–3.94 (br, PhC*H*_2_), 3.03–3.56 (COOCH_2_CH_2_CH_2_C*H*_2_), 1.94–2.14 (br, OCH_2_C*H*CH_2_), 1.14–1.73 (br, PhCC*H*_3_, COOCH_2_C*H*_2_CH_2_CH_2_, COOCH_2_CH_2_C*H*_2_CH_2_).

**Synthesis of SPU-Bisphenol A**

SPU-Bisphenol A-S was synthesized by the following method. In a two-neck round-bottomed flask, PTMG (*M*_n_ = 1000) (3.12 g, 3.12 mmol) was dried in vacuo for 1.5 h at 80 °C. Bisphenol A-diol (0.955 g, 2.77 mmol) was added. After two freeze vacuum drying cycles by benzene, *N*,*N*-dimethylacetamide (140 mL), MDI (1.81 g, 7.23 mmol), and di-*n*-butyltin dilaurate diluted with tetrahydrofuran (2 drops) were added. After stirring for 13 h at room temperature, BDO (0.170 g, 1.89 mmol) was added to the mixture under a nitrogen atmosphere. After stirring for 13 h at room temperature, the reaction was stopped by adding methanol (5 drops). The crude product was added to water and precipitated in tetrahydrofuran/water, and dried in vacuo to give a white solid (5.89 g, 97%).

^1^H NMR (500 MHz, DMSO-*d*_6_): *δ* /ppm 9.36–9.70 (br, N*H*), 7.20–7.50 (br, aromatic), 6.92–7.17 (br, aromatic), 6.62–6.87 (br, aromatic), 4.12–4.30 (br, OC*H*_2_*C*H_2_CH_2_), 3.83–4.12 (br, OCH_2_*C*H_2_C*H*_2_, COOC*H*_2_CH_2_CH_2_CH_2_), 3.68–3.83 (br, PhC*H*_2_), 3.04–3.52 (COOCH_2_CH_2_CH_2_C*H*_2_), 1.95–2.12 (br, OCH_2_C*H*CH_2_), 1.29–1.73 (br, PhCC*H*_3_, COOCH_2_C*H*_2_CH_2_CH_2_, COOCH_2_CH_2_C*H*_2_CH_2_).

**Synthesis of PCL-DABBF**

PCL-DABBF was synthesized by the following method. In a round-bottomed flask, DABBF-diol (0.132 g, 0.167 mmol) and diphenyl phosphate (83.0 mg, 0.331 mmol) were added. After freeze vacuum drying by benzene, ε-caprolactone (29.7 g, 0.260 mol) was added to the flask under a nitrogen atmosphere. The mixture was stirred until it became solid at room temperature. After dissolving the crude product in chloroform, the solution was added to methanol. Fractional precipitation in chloroform/methanol and drying in vacuo to give a pale green solid (8.37 g, 61%).

^1^H NMR (500 MHz, CDCl_3_): *δ* /ppm 7.29 (br, aromatic), 6.77–6.84 (br, aromatic), 4.24–4.29 (br, OC*H*_2_CH_2_CH_2_), 4.18–4.23 (br, OCH_2_CH_2_C*H*_2_), 4.06 (t, OC*H*_2_CH_2_CH_2_ CH_2_CH_2_), 2.31 (t, OCH_2_CH_2_CH_2_CH_2_C*H*_2_), 2.09–2.15 (br, OCH_2_C*H*_2_CH_2_), 1.60–1.70 (br, OCH_2_C*H*_2_CH_2_CH_2_CH_2_, OCH_2_CH_2_CH_2_C*H*_2_CH_2_), 1.34–1.43 (br, OCH_2_CH_2_C*H*_2_CH_2_CH_2_), 1.04–1.38 (br, tBu).

**Synthesis of PCL-DABBT**

PCL-DABBT was synthesized by the following method. In a round bottom flask, DABBT-diol (0.100 g, 0.167 mmol) and diphenyl phosphate (83.0 mg, 0.331 mmol) were added. After freeze vacuum drying by benzene, ε-caprolactone (28.6 g, 0.251 mol) was added to the flask under a nitrogen atmosphere. The mixture was stirred until it became solid at room temperature. After dissolving the crude product in chloroform, the solution was added to methanol. Fractional precipitation in chloroform/methanol and drying in vacuo to give a pale green solid (6.21 g, 43%).

^1^H NMR (500 MHz, CDCl_3_): *δ* /ppm 7.47–7.78 (br, aromatic), 7.21–7.47 (br, aromatic), 6.59-6.94 (br, aromatic), 4.24–4.29 (br, OC*H*_2_CH_2_CH_2_), 4.18–4.23 (br, OCH_2_CH_2_C*H*_2_), 4.06 (t, OC*H*_2_CH_2_CH_2_ CH_2_CH_2_), 2.31 (t, OCH_2_CH_2_CH_2_CH_2_C*H*_2_), 2.09–2.15 (br, OCH_2_C*H*_2_CH_2_), 1.60–1.70 (br, OCH_2_C*H*_2_CH_2_CH_2_CH_2_, OCH_2_CH_2_CH_2_C*H*_2_CH_2_), 1.34–1.43 (br, OCH_2_CH_2_C*H*_2_CH_2_CH_2_), 1.04–1.38 (br, tBu).

**Figure S1**. ^1^H NMR spectrum of SPU-DABBF-H (500 MHz, DMSO-*d*_6_).

**Figure S2**. ^1^H NMR spectrum of SPU-TASN-H (500 MHz, DMSO-*d*_6_).

**Figure S3**. ^1^H NMR spectrum of SPU-DABBT-H (500 MHz, DMSO-*d*_6_).

**Figure S4**. ^1^H NMR spectrum of SPU-Bisphenol A-S (500 MHz, DMSO-*d*_6_).

**Figure S5**. GPC profile of SPU-DABBF-H (RI, THF).

**Figure S6**. GPC profile of SPU-TASN-H (RI, THF).

**Figure S7**. GPC profile of SPU-DABBT-H (RI, THF).

**Figure S8**. GPC profile of SPU-DABBF-S (RI, THF).

**Figure S9**. GPC profile of SPU-TASN-S (RI, THF).

**Figure S10**. GPC profile of SPU-Bisphenol A-S (RI, THF).

**Figure S11**. DSC profile of SPU-DABBF-H.

**Figure S12**. DSC profile of SPU-TASN-H.

**Figure S13**. DSC profile of SPU-DABBT-H.

**Figure S14**. DSC profile of SPU-DABBF-S.

**Figure S15**. DSC profile of SPU-TASN-S.

**Figure S16**. DSC profile of SPU-Bisphenol A-S.

**Figure S17**. Stress-strain curves of Films 1–7 (100 mm/min, dumbbell shapes).

**Figure S18**. ^1^H NMR spectrum of PCL-DABBF (500 MHz, CDCl_3_).

**Figure S19**. ^1^H NMR spectrum of PCL-DABBT (500 MHz, CDCl_3_).

**Figure S20**. GPC profile of PCL-DABBF (RI, THF).

**Figure S21**. GPC profile of PCL-TASN (RI, THF).

**Figure S22**. GPC profile of PCL-DABBT (RI, THF).

**Figure S23**. GPC profile of PCL-Bisphenol A (RI, THF).

**Figure S24**. DSC profile of PCL-DABBF.

**Figure S25**. DSC profile of PCL-TASN.

**Figure S26**. DSC profile of PCL-DABBT.

**Figure S27**. DSC profile of PCL-Bisphenol A.


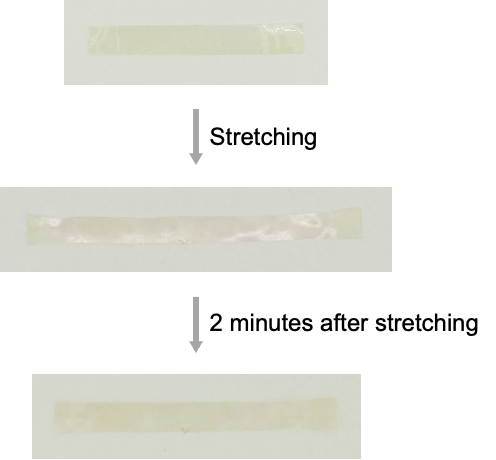


**Figure S28**. Photographs of SPU-TASN-S film before, just after, and 2 minutes after stretching.


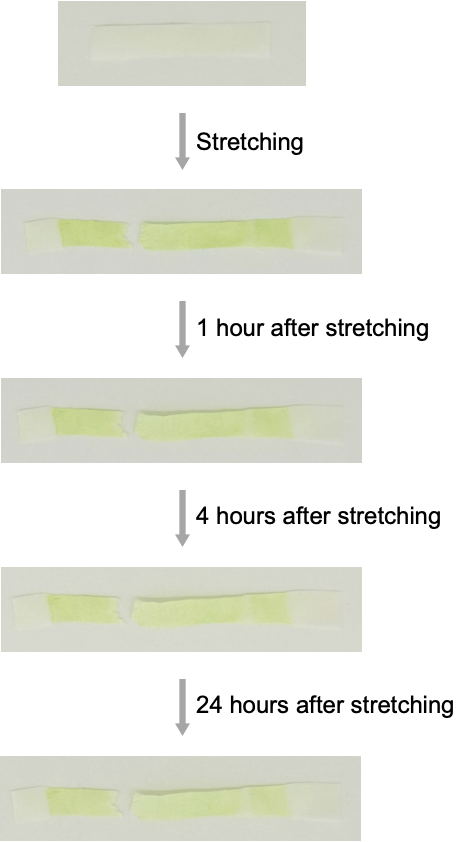


**Figure S29**. Photographs of PCL-DABBT film before, just after, 1 hour after, 4 hours after, and 24 hours after stretching.

**Figure S30**. DSC profile of Film 10 (blend film of SPU-TASN-S and PCL-DABBT).

**Figure S31**. Stress–strain curves (100 mm/min, strips) of SPU-TASN-S, PCL-DABBT, and Film 10 (blend film of SPU-TASN-S and PCL-DABBT).

**SI References**

1. K. Imato, A. Irie, T. Kosuge, T. Ohishi, M. Nishihara, A. Takahara, H. Otsuka, *Angew. Chem., Int. Ed.* **2015**, *54*, 6168.
2. T. Sumi, R. Goseki, H. Otsuka, *Chem. Commun.* **2017**, *53*, 11885.
3. K. Ishizuki, H. Oka, D. Aoki, R. Goseki, H. Otsuka, *Chem. Eur. J.* **2018**, *24*, 3170.
4. S. Kato, D. Aoki, K. Oikawa, K. Tsuchiya, K. Numata, H. Otsuka, *ACS Macro Lett.* **2021**, *10*, 623.
